# Right heart failure after left ventricular assist device: From mechanisms to treatments

**DOI:** 10.3389/fcvm.2022.1023549

**Published:** 2022-10-19

**Authors:** Claudio A. Bravo, Andrew G. Navarro, Karanpreet K. Dhaliwal, Maziar Khorsandi, Jeffrey E. Keenan, Parvathi Mudigonda, Kevin D. O'Brien, Claudius Mahr

**Affiliations:** ^1^Division of Cardiology, Department of Medicine, University of Washington, Seattle, WA, United States; ^2^School of Medicine, University of Washington, Seattle, WA, United States; ^3^Division of Cardiothoracic Surgery, Department of Surgery, University of Washington, Seattle, WA, United States

**Keywords:** right ventricular failure, right heart failure, left ventricular assist device, right ventricle, advanced heart failure

## Abstract

Left ventricular assist device (LVAD) therapy is a lifesaving option for patients with medical therapy-refractory advanced heart failure. Depending on the definition, 5–44% of people supported with an LVAD develop right heart failure (RHF), which is associated with worse outcomes. The mechanisms related to RHF include patient, surgical, and hemodynamic factors. Despite significant progress in understanding the roles of these factors and improvements in surgical techniques and LVAD technology, this complication is still a substantial cause of morbidity and mortality among LVAD patients. Additionally, specific medical therapies for this complication still are lacking, leaving cardiac transplantation or supportive management as the only options for LVAD patients who develop RHF. While significant effort has been made to create algorithms aimed at stratifying risk for RHF in patients undergoing LVAD implantation, the predictive value of these algorithms has been limited, especially when attempts at external validation have been undertaken. Perhaps one of the reasons for poor performance in external validation is related to differing definitions of RHF in external cohorts. Additionally, most research in this field has focused on RHF occurring in the early phase (i.e., ≤1 month) post LVAD implantation. However, there is emerging recognition of late-onset RHF (i.e., > 1 month post-surgery) as a significant cause of morbidity and mortality. Late-onset RHF, which likely has a unique physiology and pathogenic mechanisms, remains poorly characterized. In this review of the literature, we will describe the unique right ventricular physiology and changes elicited by LVADs that might cause both early- and late-onset RHF. Finally, we will analyze the currently available treatments for RHF, including mechanical circulatory support options and medical therapies.

## Introduction

Heart transplantation still is considered the gold-standard treatment for advanced heart failure (1–3). Yet, limited donor organ supply limits this therapy to a small proportion of those who might benefit from it. Left ventricular assist device (LVAD) therapy has emerged as a viable option for those who cannot be transplanted before an irreversible complication or death occurs. LVAD placement immediately produces lifesaving hemodynamic changes such as normalization of cardiac output and reducing left ventricular (LV) pressures (4). These changes improve end-organ function, functional capacity, and survival (4–6). The evolution of LVAD technology has led to a 2-year event-free survival of around 78% with the latest commercial LVAD iteration, the HeartMate 3 (Abbott) (7, 8).

However, due to mechanisms that remain incompletely characterized, a significant number of patients develop right heart failure (RHF) following LVAD implantation. Depending on the definition, up to 40% of people supported with LVAD develop RHF, which is associated with poor outcomes (9–12). Based on the Interagency Registry of Mechanical Circulatory Support (INTERMACS) 2020 annual report, heart failure and multisystem organ failure were among the most significant causes of death among LVAD patients, with RHF likely playing a pivotal role in many (13). Thus, the development of RHF is one of the complications observed in patients chronically supported with LVADs that limits the full potential benefit from device therapy.

The treatments and understanding of most LVAD complications have evolved over the years. However, RHF remains poorly characterized and, most importantly, lacks medical treatment options. Despite the numerous compounds developed to successfully treat the failing LV, a paucity of research on RHF, including research into the essential physiological, phenotypic, histologic, and molecular differences between the right and left ventricles, has contributed to the lack of specific pharmacological agents for RHF. This manuscript aims to review the latest literature on right ventricular (RV) physiology, RHF pathophysiology in the presence of LVAD, and options to treat this complication.

## Right ventricular physiology

The right and left ventricles work together as an interdependent system and share many similarities (14–16). Nonetheless, there are key dissimilarities, including the development of cardiomyocytes of each ventricle from different embryological progenitors, as well as differences in geometry, wall thickness, and loading conditions, that underscore critical differences between the ventricles (17, 18).

**Figure 1:**
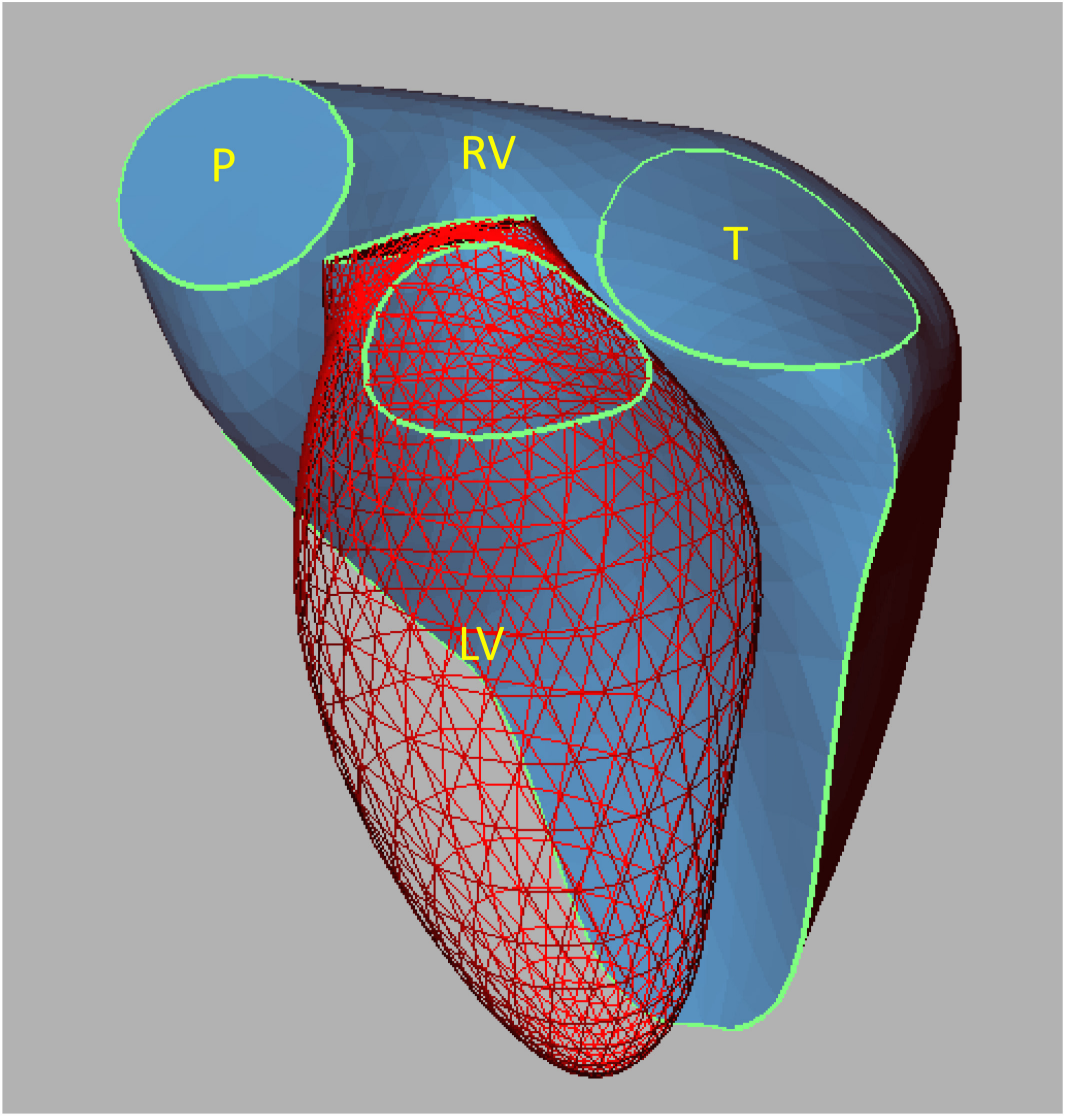
Reconstruction of a normal RV illustrating the three-dimensional relationships with the LV. The mesh surface represents the LV, and the continuous blue surface illustrates the RV. LV, left ventricle; P, pulmonary valve; RV, right ventricle; T, tricuspid valve. Reproduced from Sheehan and Redington (19).

The RV is a thin-walled crescentic-shaped chamber that wraps around the LV (Figure 1) (19–21). Since both ventricles are connected as a series of “pumps” in a normal heart, the RV must deliver the same stroke volume as the LV to maintain normal circulation. Like the LV, the RV systolic function also follows the Frank-Starling law, which governs the interaction between venous return (preload), pulmonary vascular resistance (afterload), and myocardial contractility. Compared to the LV, the RV functions at higher ventricular volumes and lower pressure and impedance, i.e., the pulmonary circulation (22, 23). This different hemodynamic environment allows the RV to provide the appropriate stroke volume, even though its myocardial mass is only one-sixth that of the LV.

**Figure 2:**
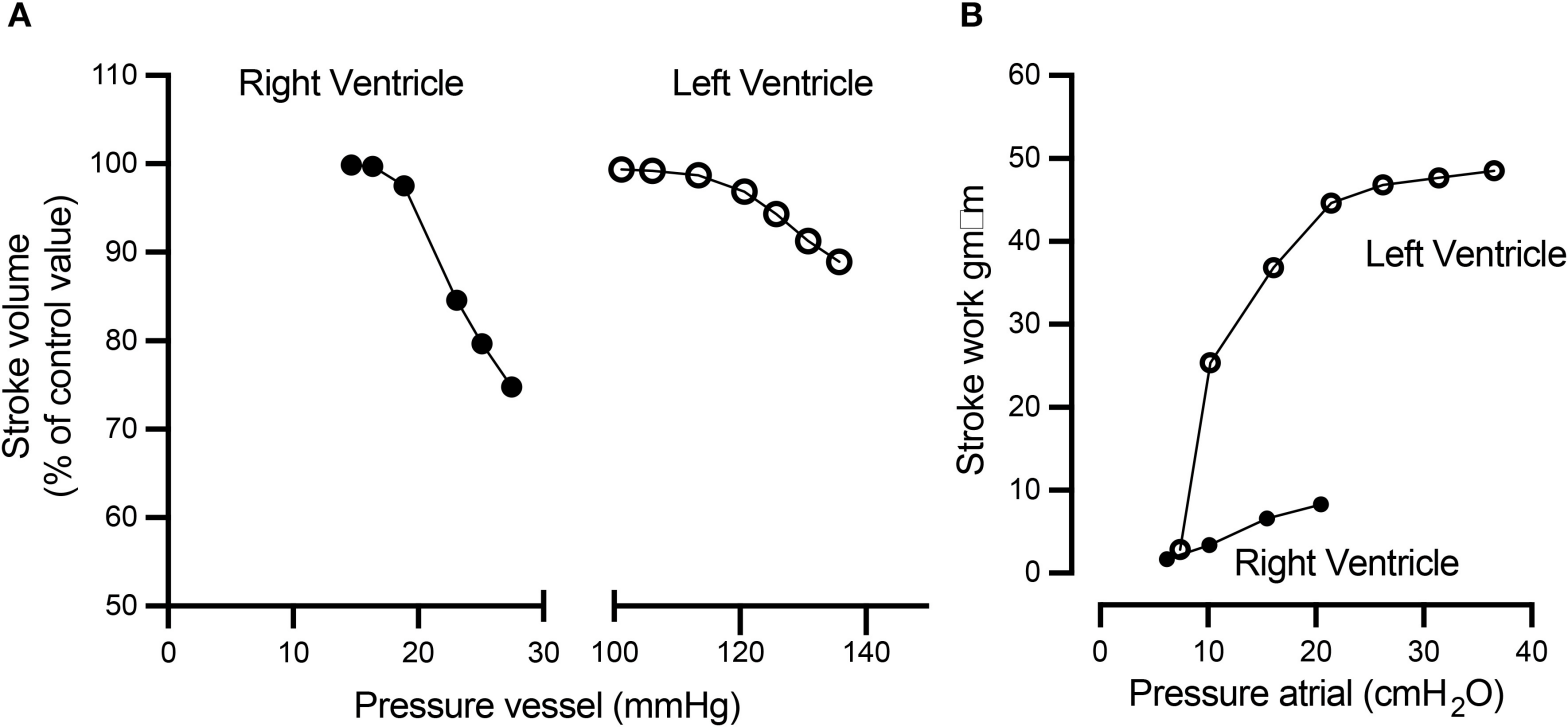
The RV and LV respond differently to increased afterload and preload. **(A)** Compared to the LV, with a 10 mmHg afterload difference (between 20 and 30 mmHg), the RV displays a rapid decline in the stroke volume, while the LV remains relatively stable. Conversely, **(B)** the LV stroke work rapidly increases as the preload increases, while the RV stroke work response is more modest to similar preload changes. This figure was published in: Braunwald (24). Reproduced by permission from Braunwald (24).

Additional unique characteristics of the RV are that it is more afterload sensitive and compliant than the LV. Increasing afterload leads to a disproportionate increase in energy expenditure and decreased efficiency of the RV as compared to the LV, resulting in a reduction of the RV stroke volume at a faster rate than the LV (Figure 2A). The higher compliance of the RV allows it to handle a large amount of blood return fluctuations without significant changes in stroke work compared to the LV (Figure 2B) (23–25).

Ventricular interdependence is crucial for the systolic and diastolic function of the RV. Ventricular interdependence refers to the size, shape, and compliance of one ventricle affecting the other through direct interaction (26, 27). Through ventricular interdependence, the LV contributes ~20–40% of the RV systolic pressure, with the RV contributing about 4–10% of the LV systolic pressure (26, 28). The interventricular septum appears to be the main contributor to ventricular interdependence. However, other factors such as muscle fibers that connect both ventricles, shared coronary blood flow, and the pericardium also play essential roles in this cooperative process (26, 29).

## Post LVAD RHF pathophysiology

**Figure 3:**
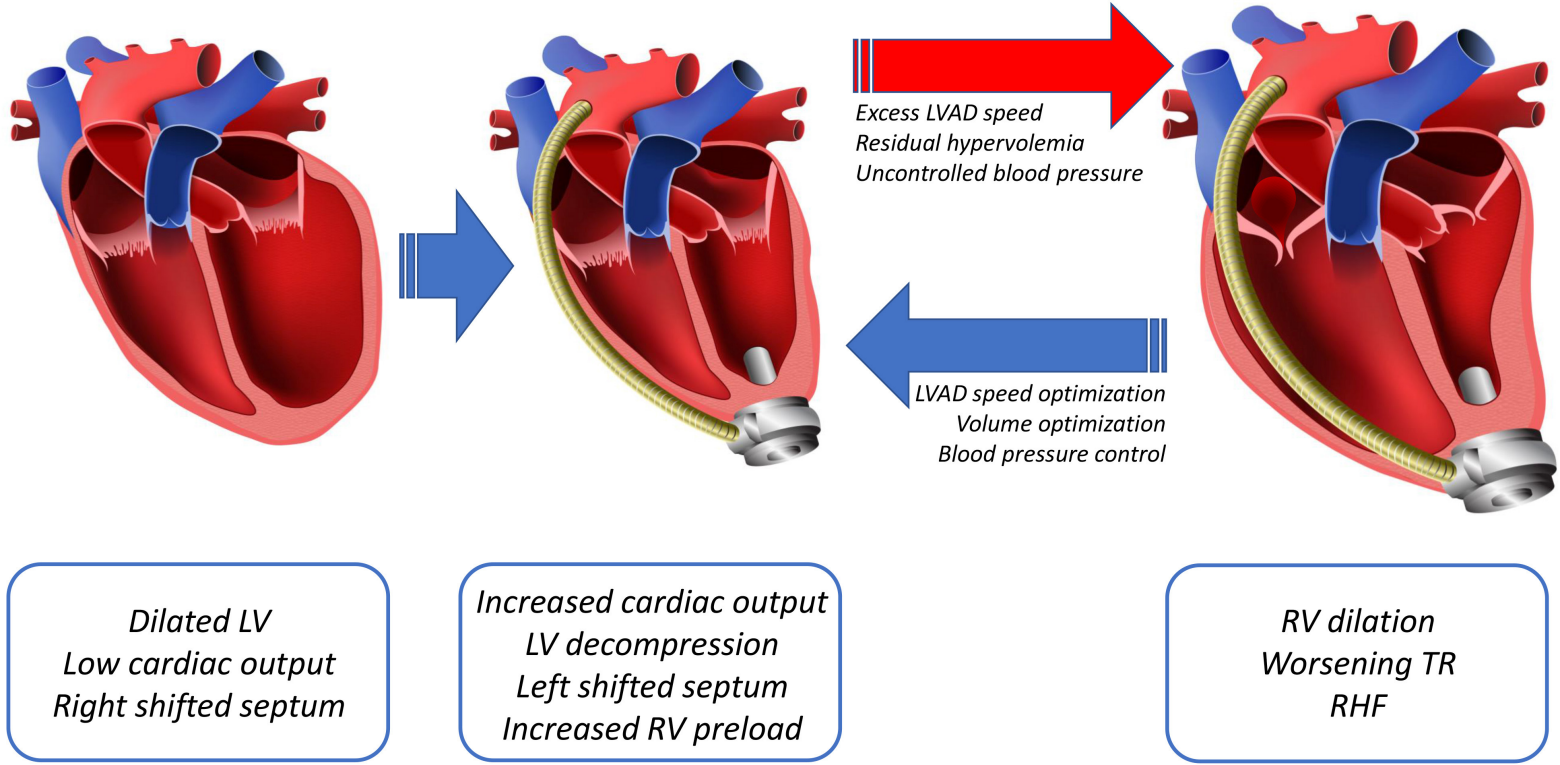
Diagram showing the main changes post LVAD, including an increased cardiac output that leads to decompression of the LV and a leftward shift of the septum with subsequently increased preload to the RV. The increased right-sided preload and a leftward shift of the septum, dilates the RV, worsening the tricuspid regurgitation (TR), resulting in RHF.

Placement of a fully functional LVAD into an advanced heart failure patient rapidly elicits favorable hemodynamic changes, including restoration of cardiac output and reductions in left ventricular and pulmonary arterial pressures (4). This new hemodynamic profile renders better end-organ perfusion, functional capacity, survival, and quality of life (4–6). The decreased pulmonary arterial pressure with LVAD, *via* immediate reduction of left-sided filling pressures and more long-term remodeling of fixed pulmonary hypertension, lowers the pulmonary vascular resistance, thereby improving right-sided afterload and RV function (4, 30–32). This LV unloading also improves RV function by decreasing functional mitral valve regurgitation and reversing an excessive shift of the septum into the RV as a result of the higher LV volume (33–35). Despite these corrective modifications of cardiogenic shock obtained with LVAD, other coexisting forces can lead to RHF (Figure 3). The pathophysiology of post-LVAD RHF is multifactorial, including post-surgical hemodynamic and geometric changes of the heart, along with perioperative and patient-related factors. Factors such as changes in preload and alterations in ventricular interdependence through pericardiotomy and interventricular septal function have been considered to play a central role in RHF pathophysiology.

### The role of preload in RHF

As LVAD support decreases right-sided afterload, it also raises preload. This can ultimately lead to a volume overloaded and more afterload-sensitive RV (36–38). The increase in RV preload is due to an increase in LV output due to LVAD support (4, 38, 39). While the RV appropriately increases the stroke volume to match the new left-sided supported cardiac output *via* a Frank-Starling mechanism, the capacity of the RV to handle a higher preload is limited by pre-existing RV functional reserve. RV distension due to a greater preload also can induce tricuspid annular dilatation, thereby worsening pre-existing tricuspid regurgitation (39–41). In essence, whenever the LVAD delivers more volume than the RV can accommodate, the result is chronic RV pressure/volume overload, eventually resulting in RHF. This occurs with excess LVAD speed, residual hypervolemia, systemic hypertension, or a combination of these factors. Tricuspid regurgitation also can be exacerbated by tricuspid valve tethering due to leftward septal shift upon LV decompression with the LVAD support, further aggravating RV pressure/volume overload (Figure 3) (42).

### The role of pericardium in RHF

One of the roles of the pericardium is to maintain biventricular morphology and interdependence, both essential features for normal RV function and geometry (26, 43). It has been widely reported that disruption of the pericardium during cardiac surgery is associated with RV dysfunction (44–46). Thus, pericardiotomy during LVAD implantation likely contributes to RHF development. Along these lines, there has been interest in using a less invasive surgical approach, such as left lateral thoracotomy, for LVAD implantation. Observational studies and the single-arm, prospective LATERAL clinical trial have suggested that with this less invasive surgical approach, LVAD implantation appears safe; and is associated with fewer blood transfusions, shorter hospital stays, and lower rates of RHF (47–52). These data suggest that preserving pericardial integrity during LVAD implantation might be protective for the RV.

### The role of the interventricular septum in RHF

The interventricular septum is a significant contributor to the RV function through its contraction and ventricular interdependence (53). Longitudinal contraction of the interventricular septum accounts for nearly 80% of normal RV function, and the oblique contraction that is responsible for the twisting motion of this chamber allows it to overcome higher afterload (54, 55). Following a cardiac surgery with pericardiotomy, the contraction pattern changes, switching to mainly transverse shortening of the interventricular septum (56). This change in the contraction pattern leads to an adaptive enhancement of transverse contractile function, maintaining a relatively normal RV function. However, these alterations make the RV more afterload sensitive. In addition to changes in interventricular contractile dynamics after pericardiotomy, the leftward septal shift due to LV decompression further limits the contribution of the interventricular septum to RV function, putting this chamber at higher risk of failure (57).

## Definitions and epidemiology of RHF

**Figure 4:**
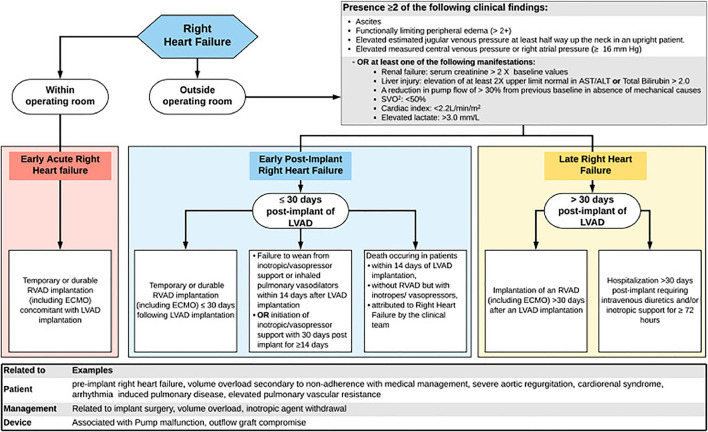
Algorithm illustrating the MCS-ARC diagnostic criteria for RHF (58). The RHF events are classified as early acute if it occurs immediately after LVAD implantation, early if it is within the first 30 days post-surgery, and late if it happened > 30 days post-surgery. Additionally, for early and late RHF, the new criteria require the presence of clinical findings or manifestations suggestive of RHF, in addition to an intervention to treat this complication. Reproduced from Kormos (58).

The definition of RHF has evolved, starting with the INTERMACS 2008, which required a central venous pressure of >18 mmHg, cardiac index < 2.0 L/min/m^2^, and a treatment for RHF such as RV mechanical circulatory support (MCS), inotrope or inhaled nitric oxide for more than a week. A version that included more variables, INTERMACS 2014, required central venous pressure of >16 mmHg or evidence of elevated central venous pressure on echocardiogram or physical exam, as well as laboratory manifestations of high central venous pressure. Finally, the 2020 consensus statement of the Mechanical Circulatory Support Academic Research Consortium (MCS-ARC) proposed a more complex and comprehensive definition of RHF. This latest definition requires signs of elevated right-sided pressures or the presence of manifestations suggestive of RHF, as well as either inotropic or mechanical intervention for this complication. Additionally, MCS-ARC RHF events are categorized as early acute, early post-implant, and late-onset, depending on the timing of RHF presentation (Figure 4) (58, 59).

Given the differing definitions and modified versions of contemporary definitions of RHF used across studies, it is challenging to understand the true burden of post-LVAD RHF and to compare its incidence across different cohorts (7, 12, 60–64). Thus, observational studies using different definitions have reported the prevalence of RHF with LVAD support ranging from 5 to 44%. However, all have reported that RHF is associated with increased morbidity, mortality, and longer hospital stay (7, 9–12, 63–67). Furthermore, most studies have focused on characterizing early onset RHF, i.e., which occurs soon after the LVAD implantation. However, there is growing evidence that RHF may manifest following patient discharge, called late-onset RHF. Late-onset RHF is less well-characterized, and it remains unknown if late-onset RHF represents part of a continuum from early RHF or a completely different entity with different etiologic factors (64, 66, 68–70).

## Prediction of RHF

There has been a significant effort to develop predictive tools for RHF in patients undergoing LVAD implantation. Pre-preoperative characteristics, echocardiographic measurements, hemodynamic parameters, and biomarkers have been associated with post-LVAD RHF incidence (71–75). Moreover, various complex scoring systems include several of those identified independent risk factors. Some of the most studied models are the Michigan RHF score system, Penn RHF risk score, Heartmate II RHF model, Utah RHF risk score, Pittsburg decision tree, CRITT score, and EuroMACS score (12, 60, 63, 76–78). Unfortunately, none of those risk scoring systems have performed as expected in external validation studies, limiting their applicability in clinical practice. One of the limitations of these studies is the heterogeneous definitions of RHF, which could partly explain the inconsistency in external validation (79).

Some hemodynamic parameters or calculations, such as high CVP, low RV stroke work index, CVP to pulmonary capillary wedge pressure ratio, pulmonary artery pulsatility index (PAPi), elevated pulmonary vascular resistance, and diastolic pulmonary gradient, have been associated with RHF (12, 61, 77, 80–84). Although, some of these parameters are widely used and cited in the clinical practice, these need further external validation using a standardized and contemporary definition of RHF.

## Treatments of RHF

Early recognition and treatment of RHF are crucial to preserve end-organ function and improve outcomes (85). Since randomized controlled clinical trials are largely lacking in this area, most evidence for RHF management is based on observational studies and personal experience, with significant variation among centers. RHF management generally comprises pulmonary artery catheter-guided therapy, volume optimization with diuretics or renal replacement treatment, pulmonary vasodilators, inotropes, heart rate or rhythm management, or mechanical RV support.

### Medical management of RHF

Volume optimization is crucial to restoring normal RV preload and afterload (86). In the setting of RHF, both low cardiac index and renal venous congestion as a consequence of an elevated central venous pressure can limit diuretic response (87). Patients refractory to diuretic treatment can benefit from ultrafiltration with renal replacement therapies (86). Additionally, in the acute setting, a reduction in pulmonary pressure by correcting hypoxia and acidosis and using pharmacologic pulmonary vasodilators can help decrease the pulmonary vascular resistance (88–91). In order to maximize RV health, it is imperative to use invasive hemodynamic optimization of LVAD speed, filling pressures, and systemic mean arterial pressure. Excess LVAD speed, residual hypervolemia, and suboptimal control of essential hypertension all contribute to chronic RV pressure/volume overload, ultimately resulting in chronic RV failure (Figure 3).

Inotropes are another palliative treatment option to provide temporary RV support by augmenting RV contractility and decreasing RV end-diastolic volume and pressure. Dobutamine and milrinone are the two most commonly utilized inotropes and have similar safety profiles regarding arrhythmogenicity (92, 93). However, milrinone has the advantage of leading to a more significant reduction in RV pressures because of its potent pulmonary and systemic vasodilatory effect compared with dobutamine (94, 95). Another relevant aspect in managing RHF is avoidance of systemic hypotension to maintain coronary artery perfusion and prevent or reduce RV ischemia (96, 97).

Atrial and ventricular tachyarrhythmias are common complications in patients with advanced heart failure. The LVAD, by unloading the LV, decreasing the adrenergic drive, and inducing reverse remodeling of the heart, has a positive impact on arrhythmogenicity. Additionally, LVAD support allows patients to remain stable and even asymptomatic while having significant atrial or ventricular arrhythmias. However, the persistence of tachyarrhythmias can be harmful to the unsupported RV contributing to the development of RHF (41, 98, 99). Therefore, although no randomized clinical trial has demonstrated that arrhythmia treatment decreases RHF incidence; it is common practice to address arrhythmia promptly and to make efforts to maintain sinus rhythm to preserve the RV function.

### Mechanical circulatory support for RHF

The timing for RV MCS placement varies among centers; some institutions offer this early on, even preventively in high-risk patients. In contrast, other hospitals offer RV MCS once medical therapy has failed. The specific support device selected also varies based on the level of support needed and the center's practice and experience.

#### Impella RP

The Impella RP (Abiomed Inc, Danvers, MA) (100) is a microaxial flow device inserted percutaneously through the femoral vein, with the distal tip positioned in the pulmonary artery. This device drains blood from the inferior vena cava and propels it into the pulmonary artery, and can provide as much as 4 L/min of flow (100, 101). Insertion of this device can be challenging due to the need to navigate the tricuspid valve and outflow tract infundibulum, and the device can be prone to migration and hemolysis.

#### Protek duo

The Protek Duo (LivaNova, London, UK) (102) is a dual lumen cannula with an inflow and an outflow limb. It is typically inserted *via* the internal jugular or subclavian veins under fluoroscopic guidance. This catheter works in conjunction with an extracorporeal centrifugal pump, the TandemHeart (LivaNova, London, UK). Advantages of this device are the relative ease of insertion and the fact that it can be inserted in an upper venous system, thereby allowing for ambulation. Disadvantages are that it also is prone to migration and, due to its diameter, can induce pulmonary valve regurgitation, thereby leading to re-circulation and ineffective RV support (103, 104).

#### Peripheral VA-ECMO

Peripheral VA-ECMO is another option for temporary RV support. However, since this strategy reduces LVAD preload by unloading the RV and increases LVAD afterload, it will negatively impact LVAD function (105). Therefore, careful LVAD/VA-ECMO adjustments must be made to avoid competition between the two devices.

#### Paracorporeal CentriMag RV assist device

The paracorporeal CentriMag RV Assist Device is a surgically implanted system that involves the placement of a right atrial or RV venous inflow cannula and a pulmonary artery arterial outflow cannula. The cannulae are connected to a paracorporeal CentriMag pump (Abbott, Chicago, IL) (106). This approach has the advantage of allowing for very effective RV support. The cannulae can be tunneled under the costal margin, allowing chest closure and ambulation. However, the most significant disadvantage of this configuration is that a sternotomy is required for insertion, and a second surgery is typically required to remove the system (107, 108).

#### Durable biventricular assist devices

There is no FDA-approved, long-term durable RV support system for LVAD patients with refractory RHF. However, small case series have reported using an LVAD system for RV support, leaving the patient with long-term dischargeable biventricular support. For right-sided device placement, the inflow cannula is placed in the right atrium or RV while the outflow graft is connected to the pulmonary artery (109, 110). Despite the feasibility of this surgical intervention, there are several concerns, including thrombus generation, especially in the venous system, and a higher risk for inflow cannula malposition and obstruction.

## Conclusion

Advances in LVAD technology have improved outcomes for patients with advanced heart failure receiving these devices. Despite these innovations, surgery-related events and the hemodynamic implications inherent to left-sided univentricular support cause cardiac morphology and dynamics changes that may eventually result in early- or late-onset RHF. There has been significant progress in managing and understanding several LVAD complications, including gastrointestinal bleeding, driveline infection, and stroke. However, RHF remains the least characterized and understood of all LVAD complications. In addition, the utilization of differing RHF definitions across studies has contributed to difficulties in thoroughly characterizing the risk factors for and pathologic mechanisms underlying this important LVAD complication. Fortunately, there is growing interest in the scientific community to fill this knowledge gap, which includes the proposal of a more inclusive and comprehensive RHF definition by the MCS-ARC. Patients with RHF after an LVAD currently receive pharmacologic treatment or MCS that maintains end-organ perfusion while the right-sided hemodynamics are optimized, allowing the RV to recover. However, we currently lack a long-term and sustainable therapy for refractory RHF, leaving only a few options for those unfortunate patients. As heart transplantation remains a very limited resource, leaving LVAD as the only option for many patients with refractory heart failure, there is an urgent need to advance research in this area.

## Author contributions

All authors listed have made a substantial, direct, and intellectual contribution to the work and approved it for publication.

## Funding

This study was funded by CB: Bristol Myers Squibb Foundation, Robert A. Winn Diversity in Clinical Trials Career Development Award, Raisbeck Gift, and R25HL145817. KO'B: NIH 5R01HL144937-03.

## Conflict of interest

Author CM is an investigator and consultant for Abbott, Abiomed, and Carmat. The remaining authors declare that the research was conducted in the absence of any commercial or financial relationships that could be construed as a potential conflict of interest.

## Publisher's note

All claims expressed in this article are solely those of the authors and do not necessarily represent those of their affiliated organizations, or those of the publisher, the editors and the reviewers. Any product that may be evaluated in this article, or claim that may be made by its manufacturer, is not guaranteed or endorsed by the publisher.
